# Overweight and obesity in type 1 diabetes equal those of the general population

**DOI:** 10.1007/s00508-018-1434-9

**Published:** 2019-01-07

**Authors:** Paul Fellinger, David Fuchs, Peter Wolf, Georg Heinze, Anton Luger, Michael Krebs, Yvonne Winhofer

**Affiliations:** 1 Division of Endocrinology and Metabolism, Department of Internal Medicine III, Medical University of Vienna, Währinger Gürtel 18–20, 1090 Vienna, Austria; 2 Section for Clinical Biometrics, Center for Medical Statistics, Informatics, and Intelligent Systems, Medical University of Vienna, Spitalgasse 23, 1090 Vienna, Austria

**Keywords:** Cardiovascular risk factors, Double diabetes, Insulin resistance, Cardiometabolic risk, Epidemiology

## Abstract

**Background:**

The obesity epidemic might affect patients with type 1 diabetes (T1DM), historically described as lean and insulin-sensitive subjects. Insulin resistance in T1DM might increase diabetic complications, especially cardiovascular disease. Therefore, the body mass index (BMI) in T1DM patients was analyzed in comparison to the general population. Furthermore, the impact of increased BMI on glycemic control and metabolic alterations was assessed.

**Methods:**

Body mass index was compared overall and among four different age groups between adult T1DM (*n* = 186), treated in the outpatient clinic between 2014 and 2016, and 15,771 individuals from the general population who took part at an Austrian health survey. Furthermore, parameters of glycemic control, lipid state, blood pressure and additional medication were compared between T1DM with a BMI under or above 27.5 kg/m^2^.

**Results:**

Patients with T1DM had significantly higher BMI values than general population (25.9 ± 4.2 kg/m^2^ vs. 25.3 ± 4.5 kg/m^2^; *p* = 0.027), controlling for age group; however, prevalence of overweight (39.8% vs. 33.1%) and obesity (14% vs. 13.8%) was not significantly different. Within the 4 age groups only T1DM patients between 30 years and 49 years old had significantly higher BMI values compared to the general population (mean difference 1.9 kg/m^2^; 95% confidence interval, CI: 0.96–2.83 kg/m^2^). In T1DM, a BMI ≥27.5 kg/m^2^ was associated with increased rates of hypertension, dyslipidemia, microalbuminuria, and increased insulin demand, whereas glycemic control was not affected.

**Conclusions:**

In contrast to common descriptions T1DM patients have a higher BMI compared to the general population. Rates of overweight and obesity in T1DM equal those of the general population. Therefore, it is concluded that the obesity epidemic has reached T1DM patients and “double diabetes” might be an entity to consider.

## Introduction

Patients with type 1 diabetes mellitus (T1DM) have traditionally been described as lean and insulin-sensitive subjects, in whom absolute insulin deficiency rather than insulin resistance is the main pathophysiological mechanism behind chronic hyperglycemia. In recent years a substantial increase in overweight and obesity in the general population across the globe was observed, talking about an obesity epidemic with around 18% of the western European population being obese [1–3]. Overweight and obesity are associated with the development of insulin resistance and consequent hyperinsulinemia, dyslipidemia and subclinical inflammation, which are all metabolic disturbances that result in the development of cardiovascular and malignant diseases [3–5]. This trend might also affect T1DM patients as suspected by previous reports [6–9].

In T1DM, especially microangiopathic complications, such as nephropathy and retinopathy, have been considered as the leading complications, causing a poor prognosis and quality of life in affected patients [10]. Fortunately, the incidence and severity of these complications have decreased in the last decades due to improved glycemic control by intensive insulin therapy [11, 12]; however, tight glycemic control is also associated with weight gain and an increased risk of hypoglycemia [12]. Weight gain in patients with T1DM might trigger the same metabolic disturbances as seen in patients with type 2 diabetes, and thus some authors suggest using the term “double diabetes”, describing the occurrence of insulin resistance in T1DM [6, 13]. Consequently, the risk for developing cardiovascular disease, the main cause of death in the population, might be even higher in these patients. As previously shown the risk of death is still doubled even in T1DM patients with good glycemic control (HbA1C <7%; <42 mmol/mol), mainly driven by cardiovascular death, and particularly women with T1DM are at special risk [14]. Hence, long-term survival in T1DM has still not reached the expected goals. Reasons for the increased mortality should be elucidated to find appropriate treatment strategies.

There is a lack of knowledge whether the rates of overweight and obesity are comparable to the general population, therefore the aim of this study was to assess the prevalence of overweight and obesity in a sample of Austrian type 1 diabetes patients in comparison with a large representative sample of the Austrian population. Furthermore, the aim was to assess whether overweight/obese patients with T1DM have worse glycemic control and metabolic alterations implicating an increased cardiovascular risk.

## Materials and methods

In this retrospective, cross sectional study all adult patients with T1DM (*n* = 186) consecutively seen between July 2014 and January 2016 in the diabetes outpatient clinic at the General Hospital in Vienna, Austria were included. If patients had multiple consultations during the observational period, the first visit during that period was used in the analyses. Type I diabetes mellitus was diagnosed based on permanent insulin treatment and positive markers for autoimmune destruction of the islet cells. The dataset of the Austrian health survey 2014 included 15,771 individuals with information about age class (5-year intervals), height, weight, and history of diabetes was provided by Statistics Austria [15]. The protocol was approved by the ethics committee of the Medical University of Vienna, protocol number 1635/2016.

### Measurements

Physical, clinical as well as laboratory data was retrieved from the medical files of the patients. Weight and height were measured using standardized methods and BMI calculated as weight (kg) divided by height squared (m^2^). Overweight and obesity were defined according to current WHO criteria as BMI 25–30 kg/m^2^ and ≥30 kg/m^2^, respectively. Due to limited number of patients in the category obesity for further analysis patients were divided into a group with a BMI less than 27.5 kg/m^2^ and a group with BMI above 27.5 kg/m^2^, in accordance with previously published protocols [16, 17].

Patient age was recorded at the day of visit and noted in full years, whereas in the health survey sample age was available only in 5‑year steps beginning with the group of 15–19 years up to more than 85 years. For further analysis, patients as well as the population were divided into 4 age groups, namely <30, 30–49, 50–69 and >69 years. The level of haemoglobin A1c (HbA1c) was used as a marker for glycemic control and further categorized in good (<7.5%; <58 mmol/mol), intermediate (7.5–9%; 58–75 mmol/mol) and poor (>9%; >75 mmol/mol) glycemic control. For evaluation of kidney disease, the albumin to creatinine ratio was measured and according to the American Diabetes Association (ADA) classification of microalbuminuria values below 30 µg/mg defined as normal, values between 30–300 µg/mg as microalbuminuria, and values >300 µg/mg as macroalbuminuria [18]. Triglycerides, low-density lipoprotein (LDL), high-density lipoprotein (HDL), and total cholesterol levels were recorded and used for analysis of the lipid metabolism. Data about daily basal as well as bolus insulin dosage were also retrieved from the medical files and for further analysis divided by body weight (in kg) as well as by body surface area which was calculated using the Mosteller formula [19].

### Statistical analysis

Data were analyzed descriptively and presented either with mean and standard deviation or median and interquartile ranges, depending on the distribution of the data. The two-sided significance level was defined as α = 0.05.

Student’s *t-*test was used for comparison of means, while the χ^2^-test was used to compare frequencies of categorical variables. The two-way analysis of variance (ANOVA) was used to compare the mean BMI between patients and the reference population while controlling for age group, and to evaluate the interaction of age and T1DM status. All analyses were performed using SPSS version 21 (SPSS, Chicago, IL, USA).

## Results

### Comparison between T1DM and the general population

Data from 186 T1DM patients were available for analyses. Of these 186 T1DM patients, 48.9% were female and 51.1% male with a mean age of 45 ± 15 years. The sample of the general Austrian population comprised 15,771 individuals, with the median age group between 45 and 49 years and an interquartile range of ±10 years. Baseline characteristics are shown in Table 1. Of the general population 4.7% reported having a diagnosis of diabetes, without further differentiation.

**Table 1:** Baseline characteristics T1DM and general population

Parameter	T1DM—mean (SD)	General population (SD)	*P*-Value
*Age (years)*	45 (15)	45–49 (10)	ns
<30 years: *N* (%)	32 (17.2)	2456 (15.6)	ns
30–49 years: *N* (%)	79 (42.5)	6034 (38.3)	
50–69 years: *N* (%)	65 (34.9)	5697 (36.1)	
>70 y: *N* (%)	10 (5.4)	1584 (10)	
*Sex m:f (%)*	51:49	44:56	ns
*BMI (kg/m* ^*2*^ *)*	25.9 (4.19)	25.3 (4.5)	0.027
<25 kg/m^2^: *N* (%)	86 (46)	8383 (53.2)	ns
25–<30 kg/m^2^: *N* (%)	74 (39.8)	5218 (33.1)	
≥30 kg/m^2^: *N* (%)	26 (14)	2170 (13.8)	
*BMI by age groups*			
<30 years (kg/m^2^)	23.7 (4.0)	23.3 (4.0)	ns
30–49 years (kg/m^2^)	26.7 (4.4)	24.8 (4.3)	<0.001
50–69 years (kg/m^2^)	26.1 (3.8)	26.4 (4.5)	ns
>70 years (kg/m^2^)	25.6 (3.0)	26.4 (4.1)	ns

*BMI* body mass index, *ns* not significant, *m* male, *f* female, *T1DM* Type 1 diabetes mellitus

**Fig. 1:**
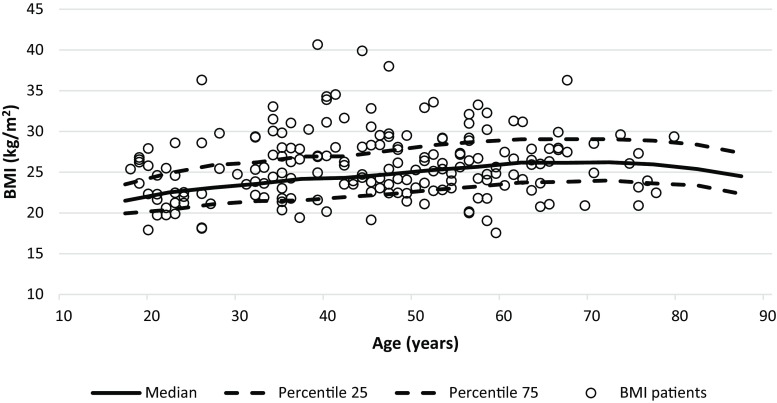
Body mass index (BMI) of each T1DM in comparison with median, 25th and 75th percentile of general population in relation to age

The mean BMI of T1DM patients was 25.9 ± 4.2 kg/m^2^ and, controlling for age group, significantly higher than the mean BMI of the general population (25.3 ± 4.5 kg/m^2^, *P* = 0.027). Fig. 1 shows a scatterplot of BMI by age overlaid with age-specific median and 25th and 75th percentiles of BMI calculated in the general population. Of T1DM patients, 64 (34.4%) had a BMI higher than the age-specific 75th percentile, while only 46.5 (25%) would be expected (*P* = 0.010). Applying the commonly used categories, 39.8% of T1DM patients and 33.1% of the general population were overweight. The prevalence of obesity was 14% in T1DM and 13.8% in the general population; however, the differences in prevalence of overweight and obesity were not significant.

Descriptive analyses of different age groups revealed that T1DM patients between 30 and 49 years had the highest BMI whereas in the general population BMI was highest in those aged 50–69 years (Table 1). In T1DM as well as in the general population the individuals younger than 30 years of age had the lowest mean BMI.

As ANOVA revealed evidence of heterogeneity in the differences in BMI between patients and the reference population across the age groups (interaction *P* = 0.017), those differences were tested separately for the four age groups. A significantly higher BMI in T1DM patients than in the reference population only in the age group between 30 and 49 years (26.7 ± 4.4 vs. 24.8 ± 4.3 kg/m^2^, corrected *p* < 0.001). This was further evidenced by the number of T1DM patients with BMI higher than the age-specific 75th percentile (observed: 35 out of 79, 44.3%; expected: 19.75, 25%; *p* < 0.001). The BMI values were not significantly different in the other age groups. Within the age group of 30–49 years it appears that female T1DM patients were responsible for the higher BMI as they had higher BMI values than their healthy counterparts (27.0 ± 5.1 vs. 23.9 ± 4.4 kg/m^2^, *p* < 0.001) in contrary to male patients where no significant difference could be found.

### Metabolic parameters in T1DM

In T1DM patients the median duration of diabetes was 17 years (interquartile range, IQR: 10–27) and mean HbA1c level was 7.7 ± 1.3% (61 ± 14 mmol/mol) with 47.3% having good, 42.5% intermediate and 10.2% poor glycemic control. The average daily basal insulin dose was 23.4 ± 11.5 IU and 30 patients had additional antidiabetic medication, like metformin.

The T1DM patients had a mean systolic blood pressure of 136.4 ± 19.7 mm Hg and a mean diastolic pressure of 81.6 ± 10.6 mm Hg, while 44% (*n* = 78) had a diagnosis of arterial hypertension. Also, 14.6% (*n* = 19) had an albumin-creatinine-ratio above 30 mg/dl and were therefore diagnosed with albuminuria, with 11.5% (*n* = 15) in the category for microalbuminuria and 3.1% (*n* = 4) meeting the criteria for macroalbuminuria. Dyslipidemia was found in 23.3% of T1DM and in 27 prescriptions of statins was recorded. Further analysis showed that T1DM had cholesterol levels of 179.9 ± 37.1 mg/dl, LDL levels of 89 ± 30 mg/dl, HDL levels of 66 ± 20 mg/dl, and triglyceride levels of 100 ± 50 mg/dl.

There was no significant BMI difference between female and male T1DM (25.6 ± 4.4 vs. 26.2 ± 3.9; *p* = ns). As shown in Table 2, T1DM with a BMI ≥27.5 kg/m^2^ had significantly higher systolic and diastolic blood pressure, higher triglyceride levels and lower HDL-cholesterol levels than T1DM with a BMI <27.5 kg/m^2^. In addition, they more often had complications like albuminuria, especially microalbuminuria. Furthermore, as expected, the basal insulin dose was significantly higher compared to T1DM with a BMI <27.5 kg/m^2^. After adjusting each patients’ basal insulin dose by dividing it through their respective body weight the difference was still significant between the two groups. In contrast neither mean HbA1c nor prevalence of good or bad glycemic control was different between the BMI groups.

**Table 2:** Patient characteristics—normal weight vs. overweight T1DM patients

Variables	BMI		*P*-value
	<27.5 kg/m^2^	≥27.5 kg/m^2^	
Women: *N* (%)	65 (50.8)	26 (44.8)	ns
Diabetes duration (years)	18.6 ± 12.7	20.8 ± 12.5	ns
Age (years)	44 ± 16	47 ± 13	ns
HbA1c (%/mmol/mol)	7.7 ± 1.4/61 ± 15	7.8 ± 1.1/62 ± 12	ns
HbA1c >7.5% (58 mmol/mol): *N* (%)	63 (49.2)	35 (60.3)	ns
Arterial hypertension: *N* (%)	72 (60)	46 (82.1)	0.004
Systolic BP (mm Hg)	133.2 ± 17.9	143.2 (21.7)	0.001
Diastolic BP (mm Hg)	80.0 ± 9.1	85.2 ± 12.8	0.002
Basal insulin dose (IU)	20.1 ± 10	30.3 ± 11.4	<0.001
Insulin (IU/m^2^/d)	10.9 ± 5.1	14.4 ± 5.1	<0.001
Insulin (IU/kg/d)	0.28 ± 0.13	0.33 ± 0.12	0.017
Creatinine (mg/dl)	0.9 ± 0.9	0.9 ± 0.2	ns
Dyslipidemia: *N* (%)	18 (16.1)	19 (40.4)	0.002
Triglyceride (mg/dl)	90.8 ± 45.3	120.6 ± 53.2	<0.001
Cholesterol (mg/dl)	182.6 ± 37	173.8 ± 37.1	ns
HDL (mg/dl)	69.5 ± 20.6	56.1 ± 13.8	<0.001
LDL (mg/dl)	92.3 ± 33.1	82.9 ± 22.6	ns
Non-HDL-cholesterol (mg/dl)	113.2 ± 36.9	118.3 ± 37.8	ns
Albuminuria: *N* (%)	7 (7.8)	12 (30)	0.001
Microalbuminuria (%)	5 (5.6)	10 (25)	0.003
Macroalbuminuria (%)	2 (2.2)	2 (5)	ns

*BMI* body mass index, *ns* not significant, *HbA1c* haemoglobin A1c, *BP* blood pressure, *HDL* high density lipoprotein, *LDL* low density lipoprotein

## Discussion

This study showed that T1DM patients have higher BMI values compared to the general Austrian population. This finding is not in agreement with the common conception that T1DM patients are lean and insulin-sensitive individuals and should raise concern as higher BMI is associated with increased risk for cardiovascular complications [20, 21]. Other studies have also shown that the prevalence of overweight and obesity in T1DM has increased over the last years, mainly due to intensive insulin treatment and changes in diet [7, 22–24]. With an average BMI of 25.9 ± 4.2 kg/m^2^ the T1DM population had a slightly higher BMI than a recent study of a large Austrian and German cohort (*n* = 31,119) which had an average BMI of 25.3 ± 6.1 kg/m^2^ [8]. Especially T1DM patients between 30 and 49 years had higher BMI values and seem to be the driving factor for higher BMI overall amongst T1DM compared to the general population, as no significantly different BMIs were found in the other age groups. The underlying reason for the significant difference in this age group is unclear. It was hypothesize that it could be associated with intensive insulin treatment, which the majority of this age group received since diagnosis. Furthermore, until recently weight management received little attention in the management of T1DM; however, the exact impact of overweight and obesity in T1DM on patient health is unclear. A recent study showed that the presence of the metabolic syndrome in T1DM was an independent risk factor for macrovascular and microvascular complications [8]. In contrast to that, other studies showed that an increase in BMI and even a BMI >30 kg/m^2^ have beneficial effects on mortality in T1DM [25, 26]. In this study it was observed that patients with an increased BMI also have a higher prevalence of microvascular as well as macrovascular risk factors. Microalbuminuria was more frequent in overweight/obese T1DM, independently of glycemic control. This observation is in accordance with other studies which showed an impact of body weight on kidney disease in T1DM as well as another study where it was shown that young healthy obese people have a higher risk of albuminuria compared to lean people [7, 27, 28]. It is well accepted that microalbuminuria is not only a sign of beginning diabetic nephropathy but is also associated with the onset of cardiovascular disease in patients with diabetes [29]; however, data in patients with double diabetes are rare, and therefore studies are needed to further investigate the exact impact of overweight in T1DM and to better understand underlying pathophysiological processes in this growing group of patients.

Interestingly, this study showed that higher BMI values are not associated with worse glycemic control, which is in contrast to other studies where significant associations between metabolic syndrome and higher HbA1c levels were found [7, 27].

Thorn et al. showed that T1DM patients with metabolic syndrome had significantly worse cholesterol, triglyceride, as well as HDL levels [7]. In this study, however, overweight T1DM had only significant increased triglyceride and lower HDL levels, showing the typical dyslipidemia of patients with metabolic syndrome.

### Limitations

This study has several limitations, including that the design was retrospective and carried out in a single center. Another limitation is that data regarding waist and hip circumference were missing and therefore it was not possible to calculate metabolic syndrome with the currently used criteria.

## Conclusion

Te results of the study showed that the obesity-epidemic has also reached patients with T1DM, who have higher BMI values compared to the general population, especially between 30 and 49 years old. Insulin resistance in these patients might not only increase insulin demand, but also the risk for cardiometabolic complications. Further research is needed to assess the additional consequences of obesity in T1DM and to develop better methods and strategies for recognizing this problem at an early stage to prevent further negative consequences.
